# Trends in mortality due to non-communicable diseases in the Brazilian adult population: national and subnational estimates and projections for 2030

**DOI:** 10.1186/s12963-020-00216-1

**Published:** 2020-09-30

**Authors:** Deborah Carvalho Malta, Bruce Bartholow Duncan, Maria Inês Schmidt, Renato Teixeira, Antonio Luiz Pinho Ribeiro, Mariana Santos Felisbino-Mendes, Ísis Eloah Machado, Gustavo Velasquez-Melendez, Luisa Campos Caldeira Brant, Diego Augusto Santos Silva, Valéria Maria de Azeredo Passos, Bruno R Nascimento, Ewerton Cousin, Scott Glenn, Mohsen Naghavi

**Affiliations:** 1 Nursing School, Department of Maternal and Child Nursing and Public Health, Universidade Federal de Minas Gerais, Belo Horizonte, Brazil; 2 Postgraduate Program in Epidemiology and Hospital das Clínicas, Universidade Federal do Rio Grande do Sul, Porto Alegre, Brazil; 3 School of Medicine, Graduate Program in Public Health, Universidade Federal de Minas Gerais, Belo Horizonte, Brazil; 4 School of Medicine, Hospital das Clínicas,Telehealth Center, Universidade Federal de Minas Gerais, Belo Horizonte, Brazil; 5 School of Medicine, Department of Internal Medicine, Universidade Federal de Minas Gerais, Belo Horizonte, Brazil; 6 School of Medicine, Department of Family Medicine, Mental and Public Health, Universidade Federal de Ouro Preto, Ouro Preto, Brazil; 7 Universidade Federal de Santa Catarina, Florianópolis, Brazil; 8 Faculdade de Ciências Médicas de Minas Gerais, Belo Horizonte, Brazil; 9 Institute for Health Metrics and Evaluation, University of Washington, Seattle, USA

**Keywords:** Global burden of disease, Non-communicable diseases, Mortality, Disability-adjusted life years, Brazil, Sustainable Development Goals

## Abstract

**Background:**

Monitoring and reducing premature mortality due to non-communicable diseases (NCDs) is a global priority of Agenda 2030. This study aimed to describe the mortality trends and disability-adjusted life years (DALYs) lost due to NCDs between 1990 and 2017 for Brazil and to project those for 2030 as well as the risk factors (RFs) attributed deaths according to estimates of the Global Burden of Disease Study.

**Methods:**

We analyzed cardiovascular diseases, chronic respiratory diseases, neoplasms, and diabetes, and compared the mortality rates in 1990 and 2017 for all of Brazil and states. The study used the definition of premature mortality (30–69 years) that is used by the World Health Organization. The number of deaths, mortality rates, DALYs, and years of life lost (YLL) were used to compare 1990 and 2017. We analyzed the YLL for NCDs attributable to RFs.

**Results:**

There was a reduction of 35.3% from 509.1 deaths/100,000 inhabitants (1990) to 329.6 deaths/100,000 inhabitants due to NCDs in 2017. The DALY rate decreased by 33.6%, and the YLL rate decreased by 36.0%. There were reductions in NCDs rates in all 27 states. The main RFs related to premature deaths by NCDs in 2017 among women were high body mass index (BMI), dietary risks, high systolic blood pressure, and among men, dietary risks, high systolic blood pressure, tobacco, and high BMI. Trends in mortality rates due to NCDs declined during the study period; however, after 2015, the curve reversed, and rates fluctuated and tended to increase.

**Conclusion:**

Our findings highlighted a decline in premature mortality rates from NCDs nationwide and in all states. There was a greater reduction in deaths from cardiovascular diseases, followed by respiratory diseases, and we observed a minor reduction for those from diabetes and neoplasms. The observed fluctuations in mortality rates over the last 3 years indicate that if no further action is taken, we may not achieve the NCD Sustainable Development Goals. These findings draw attention to the consequences of austerity measures in a socially unequal setting with great regional disparities in which the majority of the population is dependent on state social policies.

## Background

Non-communicable diseases (NCDs) represent a threat to global health and socioeconomic development. The World Health Organization (WHO) estimates that approximately 40 million deaths occur annually due to this group of diseases [1, 2]. More than 80% of these deaths occur in low- and middle-income countries (LMICs) and a third occur among individuals younger than 60 years of age, while in high-income countries, this proportion is 13% [2].

NCDs also produce significant indirect costs to society due to the reduction in productivity, loss of working days, and losses in the production sector in addition to the adverse effects on the quality of life of the affected people [3], making it imperative to invest in confronting NCDs to achieve the Sustainable Development Goals [4].

Premature deaths (30 to 69 years) due to NCDs usually affect individuals with lower income and education who are more exposed to risk factors and have reduced access to information and health services, which contributes to even greater social inequality [1, 3–5]. Studies of the impact of the financial crisis of 2008 in Europe and the fiscal austerity measures implemented in many countries, including Brazil, showed important effects on public health expenditures and worsening physical health and mental health indicators in addition to an increase in cardiovascular diseases and other NCDs [6–9].

In Brazil, as in other countries, NCDs also constitute a health problem of greater magnitude that corresponds to 75% of the causes of deaths [10]. In 2011, the Strategic Action Plan for Tackling Chronic Non-Communicable Diseases in Brazil (2011–2022) was based on three axes: (I) vigilance, information, evaluation, and monitoring; (II) health promotion; and (III) integral care. The plan established goals to reduce risk factors and premature mortality by NCDs, which became fundamental for monitoring the targeting of these goals [11]. This plan occurred within and through a unified health system (Sistema Único de Saúde [SUS]) that was implemented in 1990 to meet the considerable challenge of achieving universal access to healthcare services for the population of a highly unequal country such as Brazil [9]. Despite its consistent progress towards health access and inequality reduction, even with allocation of few resources by the government, SUS has improved several health outcomes and health systems based on the principles of universality, equity, integrality, decentralization, and community engagement [9]. More recently, SUS has faced many setbacks, especially due to the sociopolitical and economic positions of the new government, especially in relation to austerity and underfunding, which has weakened advances and reversed achievements [9].

Considering the severity of the NCDs and their impact on the health systems and society, in 2013, the WHO approved the Global Action Plan for the Prevention and Control of NCDs 2013–2020 [5]. In 2015, the WHO also included the goal of reducing premature deaths from NCDs in individuals between 30 and 69 years of age as part of the 2030 Agenda for Sustainable Development, which has been signed by Brazil [4].

The present study analyzed the mortality rates from NCDs in individuals between 30 and 69 years of age in Brazil and in the states of Brazil between 1990 and 2017, projected the mortality trends for 2030, and projected the risk factors that caused these deaths according to estimates of the Global Burden of Disease Study.

## Methods

The estimates of the Global Burden of Disease (GBD) Study from the *Institute of Health Metrics and Evaluation* (IHME) were used. This study is a systematic scientific effort to create indicators capable of measuring the magnitude of health loss due to diseases, injuries, and risk factors among countries and subnational levels by age, sex, and time. In 2017, the study analyzed data from 359 diseases and injuries, 1793 consequences, and 84 risk factors by age and sex. Detailed descriptions of the methodology and approach of the GBD in 2017 can be found in other publications [12–15].

For the estimates presented here, the GBD 2017 used a large number of data sources from Brazil. The population was estimated from data from the Brazilian Institute of Geography and Statistics (IBGE) [16], and for mortality, the main information source was the Information System about Mortality (SIM) [17] of the Brazilian Ministry of Health. For all Brazilian states, the quality of data was considered high and equivalent to that of high-income countries [18]. However, even though it is considered a developed system, as with all mortality data sources used in GBD studies, SIM went through the data treatment process to standardize and improve the quality of its data. One of the most important steps in the process is the redistribution of the underlying causes that are defined as garbage codes. Deaths attributed to causes that should not be considered an underlying cause of death because they are non-specific, have incomplete diagnosis, or did not allow adequate identification of the actions for the prevention, and control of diseases are called garbage codes [18, 19]. Causes such as heart failure, septicemia, and renal failure do not define the disease that caused death and should be redistributed into basic non-*garbage* death causes for each age-sex-year according to the GBD-specific redistribution algorithms.

For the analysis of risk factors, the main data sources used by the GBD were representative surveys of the Brazilian population; these sources included the National Health Survey (NHS), the telephone survey surveillance system for risk and protective factors for chronic diseases (VIGITEL), and the National Student Health Survey (from the Portuguese: PeNSE) [20, 21].

The contribution of the risk factors (RF) for mortality due to the NCDs was estimated using a comparative risk assessment in which the health outcomes were compared with those that would have been observed in a counterfactual exposition group where nobody was exposed [14]. The GBD study uses CODEm, a simulation model for risk factors and attributable deaths, that estimates indicators by age, sex, country, state, year, and cause; in other words, CODEm is an analytical tool that tests many possible statistical models of causes of death and creates a group of combined models that offer the best predictive development [22]. DisMod-MR 2.1 software (World Health Organization©, Genebra, Switzerland) [22], which is a metaregression tool, was used to derive the simultaneous incidence, prevalence, incapacity, and mortality estimates attributed to the risk factors [14, 20]. Modeling details are described in other studies [14, 22].

This study used the concepts of premature mortality that are used by the WHO and UN in the Global Action Plan for the Prevention and Control of NCDs (2013) [5] and in the Sustainable Development Goals (SDGs) for NCDs (2015) [4], respectively, which consider premature death to occur between 30 and 69 years of age. For the calculation of the death rates, we used the population for this age group that was estimated by the GBD [12]. In 2017, the GBD estimated a population of 212 million for Brazil in 2017, and the 30- to 69-year group represented 48% of that population, which means that it included more than 100 million inhabitants.

The following metrics were used to describe the burden of disease: the absolute number of deaths, mortality rates, disability-adjusted life years (DALYs), and years of life lost (YLLs); these were compared between 1990 and 2017 and used to describe the percentage of change in this period.

We analyzed the premature mortality rates between the ages of 30 and 69 for the following NCDs: cardiovascular diseases (I00-I99), chronic respiratory diseases (J30-J98), neoplasms (C00-C97), and diabetes mellitus (E10-E14), comparing the deaths and the NCD mortality rates, and these main groups of diseases between 1990 and 2017 and the percentage of change in the period for Brazil and the states.

The fraction of the burden of NCDs (DALYs) in Brazil that could be attributed to the risk factors was analyzed. The GBD study presents the risk factors in four levels of disaggregation: level 1 is the most aggregated and level 4 is the most disaggregated. The risk factors at level 1 are behavioral, metabolic, and environmental, while at level 2, which is more disaggregated than level 1, the definitions include factors such as malnutrition, high blood pressure, and air pollution. We used level 2 to present the RFs in a more aggregated way. For each main RF group, the DALYs attributable to the different groups of diseases and were estimated by sex for 1990 and 2017.

The Agenda 2030 set the goal of reducing premature mortality from NCDs by 30% between 2015 and 2030, which meant going from 323.3 deaths per 100,000 inhabitants to fewer than 226.3 in Brazil. By considering SDG Indicator 3.4.1, which is the age-standardized death rate due NCDs (to cardiovascular diseases (CVD), cancers, diabetes, and chronic respiratory diseases) among populations aged 30 to 70years (per 100,000), projections were made until 2030 [4]. Due to the mortality rates that have shown a possible increase in recent years, different future scenarios were predicted: (a) using the estimates of the GBD Study to calculate all of the SDG indicators [23]; (b) using linear regression and adopting the same behavior as that of the mortality rates between 2000 and 2017, which was the basis for the calculation of the Strategic Action Plan for Tackling Chronic Non-Communicable Diseases in the country [11]; (c) using linear regression and adopting the same behavior as the mortality rates between 2010 and 2017, which was the first year of the Strategic Action Plan for Tackling Chronic Non-Communicable Diseases in the country [11]; and (d) using linear regression and adopting the same behavior for the mortality rates as that between 2015 and 2017, which was the first year of the fluctuation in mortality rates.

The results were presented with their respective uncertainty intervals (UI), which reflected the uncertainty of the estimates of the parameters for each state and period of study. More methodological details can be found in other publications [12–14, 22].

The Brazil GBD study was approved by the Ethics Committee in Research of the Federal University of Minas Gerais (COEP/UFMG); the approval number was CAAE 62803316.7.0000.5149.

## Results

**Fig. 1:**
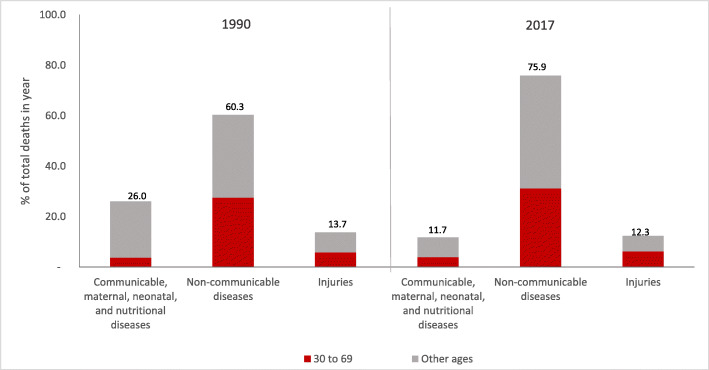
Proportional mortality according to groups of causes and proportion of deaths in the adult population between 30 and 69 years old in Brazil in 1990 and 2017. Legend: Proportion of total deaths according to communicable, maternal, neonatal, and nutritional diseases; non-communcable diseases; and injuries and proportion of deaths in the adult population between 30 and 69 years old and other ages in Brazil in 1990 and 2017

Figure 1 shows the distribution grouped by cause of death in Brazil according to the level 1 disaggregation, both for sexes and the entire population. In 1990, the proportional mortality due to NCDs for all ages corresponded to 60.3% (UI 59.8–62.5%) of deaths; the external causes, 13.7% (UI 13.5–15.1); and the group of neonatal nutritional and maternal deaths, 26% (UI 23.8–26.0). In 2017, the proportional mortality from NCDs increased to 75.9% (UI 75.6–77.2), and the deaths by external causes decreased to 11.7% (UI 10.8–11.5). The proportion of premature deaths due to NCDs (30 to 69 years of age) remained stable at 27% in 1990 and 28% in 2017.

In 2017, the age group of 30 to 69 years presented 41.3% (556.639) of all deaths, and the premature deaths from the four NCDs were 58.9% (327,954 deaths). Although between 1990 and 2017, the absolute numbers of deaths increased by 50.4%, the standardized mortality rates from NCDs decreased by 35.3%. The reduction also occurred in the DALY and YLL rates (Table 1).

**Table 1:** Number of deaths and age-standardized mortality, DALY, and YLL rates, in the population between 30 and 69 years of age in both sexes in Brazil in 1990 and 2017

Cause of death	Number of deaths			Age-standardized death rates (per 100,000)			Age-standardized DALY (per 100,000)			Age-standardized YLL (per 100,000)		
	1990	2017	% change	1990	2017	% change	1990	2017	% change	1990	2017	% change
All causes	356,191.5	556,639.0	56.3	799.4	557.5	− 30.3	41,795.8	32,909.9	− 21.3	27,086.4	18,758.8	− 30.7
NCD*	218,066.0	327,954.8	50.4	509.1	329.6	− 35.2	17,886.2	11,884.7	− 33.6	16,006.0	10,251.6	− 36.0
Cardiovascular diseases	126,247.4	152,382.5	20.7	294.3	153.2	− 47.8	9785.7	5209.0	− 46.8	9,281.9	4,750.1	− 48.8
Chronic respiratory diseases	15,898.2	22,074.6	38.8	38.0	22.3	− 41.3	1731.3	1081.4	− 37.5	1,122.6	657.0	− 41.5
Diabetes mellitus	12,122.6	23,295.3	92.2	28.7	23.5	− 17.8	1555.9	1344.9	− 13.6	873.4	702.0	− 19.6
Neoplasms	63,797.8	130,202.3	104.1	148.1	130.5	− 11.8	4813.3	4249.4	− 11.7	4,728.1	4,142.5	− 12.4

Legend: Number of deaths, age-standardized mortality rates, age-standardized DALY rates and age-standardized YLL rates, in the population between 30 and 69 years of age in both sexes for all causes, non-communicable diseases (NCD), and main non-communicable diseases separately in Brazil in 1990 and 2017.*NCD = cardiovascular diseases, neoplasms, diabetes, and chronic respiratory diseases

Table 1 also shows important reductions in the age-standardized mortality rates among the four main categories of NCDs. During the study period, the mortality caused by CVDs decreased by 47.9%, and the mortality caused by chronic respiratory diseases, diabetes mellitus, and neoplasms decreased by 41.3%, 18%, and 11.9%, respectively. The DALY and YLL rates for these categories experienced similar reductions.

Table 2 shows the standardized mortality rates by age, for those between 30 and 69 years of age, by NCD, by sex, according to causes of death selected for all Brazil and the states of Brazil, and for 1990 and 2017. In most of the states, a reduction of rates for NCDs, CVDs, and chronic respiratory disease occurred. Greater reductions in mortality caused by CVDs were observed in specific states, with the largest change in the Federal District (FD) (− 60.3% UI − 62.3%; − 57.0%), which also had the largest reduction in chronic respiratory disease (− 55.1%; UI − 59.4%; − 49.9%). For diabetes and neoplasms, the patterns varied by state, with some presenting increases and others decreasing. For diabetes mellitus, there was a larger reduction in the FD (− 43.1%; UI − 50.0%; − 35.6%) and an increase in Pará (45.5%; UI 28.9%; 64.6%). The mortality due to neoplasms had larger reductions in the FD (− 28.8% UI − 32.1%, 25.2%) and a larger increase in Rio Grande do Norte (18.9%; UI − 9.3%; 31.1%) and remained stable in most states.

**Table 2:** Rate of premature mortality by non-communicable diseases standardized by age in the population between 30 and 69 years of age for both sexes and according to the selected causes of death in Brazil and its states in 1990 and 2017

Local	Cardiovascular diseases			Chronic respiratory diseases			Diabetes mellitus			Neoplasms			NCD*		
	1990	2017	% change	1990	2017	% change	1990	2017	% change	1990	2017	% change	1990	2017	% change
Brazil	294.3 (290.8; 298)	153.2 (151.2; 155.2)	− 47.9 (− 48.8; − 47.1)	38 (37.2; 38.9)	22.3 (21.7; 22.9)	− 41.3 (− 43.4; − 39.3)	28.7 (27.8; 29.6)	23.5 (22.9; 24.1)	− 18 (− 21.2; − 14.9)	148.1 (145.4; 151.1)	130.5 (128.5; 134.3)	− 11.9 (− 13.7; − 9.5)	509.1 (505.2; 512.7)	329.6 (326.3; 333.8)	− 35.3 (− 36; − 34.5)
Acre	223.5 (215.9; 230.8)	127.2 (121.3; 133.2)	− 43.1 (− 46.3; − 39.7)	41 (38.3; 44)	30.2 (27.9; 32.7)	− 26.5 (− 34.1; − 18.2)	21.8 (20.1; 23.7)	22.8 (20.8; 24.8)	4.4 (− 7.4; 18.4)	120.4 (115; 129.7)	113.1 (107.5; 120.7)	− 6.1 (− 11; − 0.2)	406.8 (397; 419)	293.3 (283.6; 303.7)	− 27.9 (− 30.7; − 25)
Alagoas	271 (262.6; 279.5)	194.1 (186.6; 202.2)	− 28.4 (− 31.9; − 24.7)	33.9 (31.2; 37.1)	23.1 (21.4; 24.9)	− 31.8 (− 39.5; − 23.5)	37.7 (34.5; 41)	45.2 (41.6; 49)	20 (6.1; 35.8)	93.1 (88.8; 97.9)	102.7 (98; 109.3)	10.3 (4; 18)	435.7 (427.6; 443.8)	365.2 (353.9; 377.1)	− 16.2 (− 19.1; − 13.2)
Amapá	183.4 (177.8;189.5)	134.6 (128.8; 140.5)	− 26.6 (− 30.8; − 22.4)	24 (22.1; 25.8)	19.6 (17.8; 21.4)	− 18.2 (− 27.7; − 7.8)	19.1 (17.5; 20.8)	24.2 (22.1; 26.5)	26.7 (10; 44.1)	129.1 (123.9; 136.6)	128.2 (122.5; 134.6)	− 0.7 (− 6.2; 5.4)	355.6 (346.6; 365.1)	306.6 (297.5; 316.3)	− 13.8 − 16.9; − 10.2)
Amazonas	192.5 (184.1; 200.6)	112 (107.3; 117.1)	− 41.8 (− 45.4; − 37.8)	26.2 (24.3; 28.1)	17.2 (16; 18.6)	− 34.3 (− 40.8; − 27)	22.4 (20.6; 24.4)	25.5 (23.4; 27.8)	13.8 (1.4; 27.5)	139.6 (133; 146.7)	134.6 (129.3; 139.5)	− 3.6 (− 9; 2.3)	380.8 (366.1; 394.6)	289.3 (279.9; 298.5)	− 24 − 27.6; − 20.2)
Bahia	229.2 (216.2; 242.8)	152.6 (146.9; 158.5)	− 33.4 (− 37.9; − 28.7)	30 (27.6; 32.5)	23.3 (21.7; 25)	− 22.3 (− 30.2; − 13.2)	31.9 (29.1; 34.9)	32.4 (29.8; 34.9)	1.5 (− 9.6; 13.9)	102.2 (95.8; 110)	120.6 (116.3; 125)	18 (9.6; 27.1)	393.3 (371.9; 415.1)	328.9 (320.6; 337.6)	− 16.4 (− 21.1; − 11)
Ceará	160.8 (147.7; 173.5)	119.1 (114.5; 124.3)	− 25.9 (− 31.7; − 19.2)	23.1 (20.5; 26.3)	14.9 (13.9; 16)	− 35.4 (− 44.8; − 26.1)	16.5 (14.7; 18.5)	18.1 (16.6; 19.8)	9.6 (− 5; 26.2)	122.3 (113.9; 130.7)	114.2 (110.4; 117.9)	− 6.7 (− 13.2; 1.1)	322.7 (304.5; 341.4)	266.3 (259.1; 274.6)	− 17.5 (− 22.3; − 12.4)
Distrito Federal	271.7 (265; 278.7)	107.6 (102.3; 113.3)	− 60.4 (− 62.4; − 58.1)	29.5 (27.6; 31.5)	13.3 (12.2; 14.5)	− 55.1 (− 59.4; − 50)	24.7 (22.7; 26.7)	14.1 (12.7; 15.4)	− 43 (− 49.8; − 35.6)	157.2 (152.2; 161.4)	111.9 (107.3; 116.8)	− 28.8 (− 32.1; − 25.2)	483.2 (475.5; 490.6)	246.8 (238.2; 255.7)	− 48.9 (− 50.8; − 46.8)
Espírito Santo	296.4 (289.9; 303.2)	139.4 (134; 144.7)	− 53 (− 55.1; − 50.9)	31.6 (29.8; 33.5)	16.3 (15.1;17.5)	− 48.4 (− 53; − 43.3)	22.7 (21; 24.4)	20 (18.3; 21.7)	− 12 (− 21.4; − 2.5)	137.4 (133.5; 142.2)	117.4 (112.7; 122.8)	− 14.6 (− 18.5; − 10.7)	488.1 (481.5; 494.8)	293 (283.5; 302.4)	− 40 (− 42; − 38.1)
Goiás	261.4 (252.6; 268.9)	147.4 (141.3; 153.3)	− 43.6 (− 46.2; − 40.8)	46.7 (43.9; 49.6)	24.8 (23.1; 26.7)	− 46.8 (− 51.4; − 42)	18.7 (17.2; 20.2)	19.9 (18.3; 21.7)	6.6 (− 5.1;20.1)	131.9 (128.1; 135.7)	114.3 (110.4; 119)	− 13.4 (− 17.3; − 9.2)	458.7 (449.3; 467.3)	306.4 (297.9; 315.7)	− 33.2 (− 35.4; − 30.9)
Maranhão	267.2 (248.3; 287.1)	163.8 (156; 171.6)	− 38.7 (− 43.5; − 33.6)	29.3 (25.5; 35.4)	15.4 (14.2; 16.7)	− 47.3 (− 55.6; − 39.1)	35.2 (30.7; 39.4)	35.5 (32.6; 38.8)	1.1 (− 12.9; 17.5)	114.8 (103.5; 125.4)	96.7 (91.9; 101.6)	− 15.8 (− 23.5; − 8.1)	446.5 (419; 474.3)	311.4 (299.4; 323.7)	− 30.3 (− 35; − 25.2)
Mato Grosso	254.4 (238.5; 272.2)	138.7 (132.8; 144.7)	− 45.5 (− 49.8; − 41)	31.2 (28.4; 34.2)	22.4 (20.7; 24.2)	− 28.4 (− 36.2; − 19.6)	20.2 (18.3; 22.2)	24.6 (22.6; 26.9)	21.3 (7.3; 37.8)	119.9 (111.5; 129.9)	111.5 (107.1; 116.9)	− 7 (− 13.9; 0.5)	425.8 (400.1; 452.6)	297.1 (287.8; 307.9)	− 30.2 (− 34.9; − 25.2)
Mato Grosso do Sul	293.8 (284.4; 302.1)	170.4 (164.1; 177.6)	− 42 (− 44.8; − 38.8)	32.5 (30.5; 34.8)	21.8 (20.2; 23.5)	− 32.9 (− 39.4; − 25.7)	19.3 (17.8; 20.9)	21 (19.3; 22.9)	9.1 (− 2.7; 22.9)	131.4 (126.2; 136.5)	126 (120.6; 132.8)	− 4.1 (− 9.6; 1.6)	477 (463.5; 487.4)	339.3 (328.4; 350.7)	− 28.9 (− 31.4; − 25.8)
Minas Gerais	304.9 (298.4; 311.4)	140.4 (135.6; 145.5)	− 53.9 (− 55.7; − 52)	43 (40.7; 45.5)	20.3 (19; 21.7)	− 52.7 (− 56.5; − 48.7)	27.3 (25.4; 29.3)	20.1 (18.5; 21.8)	− 26.6 (− 34.2; − 18.1)	136 (132.7; 139.8)	124.8 (120.9; 129.2)	− 8.2 (− 11.8; − 4.4)	511.2 (505.1; 517.1)	305.6 (298.5; 314)	− 40.2 (− 41.6; − 38.6)
Pará	218.1 (207.7; 228.5)	142.2 (136; 148.6)	− 34.8 (− 39.1; − 30.3)	27.9 (25.7; 29.9)	21.6 (20.1; 23.3)	− 22.6 (− 30; − 13.5)	20 (18.4; 21.7)	29 (26.7; 31.6)	44.9 (28.5; 63.7)	118.5 (112; 126.7)	114.3 (109.8; 120.1)	− 3.6 (− 9; 2.6)	384.6 (367.8; 401.2)	307.1 (297.3;317.3)	− 20.2 (− 24.4; − 16)
Paraíba	209.6 (197; 222)	163.4 (151; 175.6)	− 22 (− 29.6; − 14)	25.5 (23; 28.7)	18.6 (16.8; 20.4)	− 27.4 (− 37.5; − 16.6)	30 (26.9; 33.4)	33.6 (30.4; 37.1)	12 (− 4.2; 29.9)	110.6 (104.1; 116.9)	113.6 (105.1; 122.4)	2.7 (− 7.4; 14.1)	375.7 (361.8; 390.9)	329.2 (306.5; 352.9)	− 12.4 (− 19.2; − 5.5)
Paraná	329.9 (323.7; 336.4)	144.3 (138.9; 149.9)	− 56.3 (− 58.1; − 54.3)	48.8 (46.1; 51.6)	26.7 (24.9; 28.6)	− 45.3 (− 50.1; − 40)	23.3 (21.7; 25)	23.9 (22.1; 25.8)	2.6 (− 7.3; 14.9)	161.5 (157.8; 165.8)	141.2 (136.4; 145.9)	− 12.6 (− 16; − 9)	563.5 (558.4; 568.8)	336.2 (327.6; 345.1)	− 40.3 (− 41.9; − 38.7)
Pernambuco	290.8 (284; 297.6)	190.6 (184.1; 197.6)	− 34.5 (− 37; − 31.5)	32.9 (30.8; 35.1)	33.1 (30.7; 35.5)	0.5 (− 8; 9.9)	38.4 (35.4; 41.4)	34.5 (31.7; 37.4)	− 10.1 (− 19.3;0.4)	119.4 (116.1; 123)	125.9 (121.7; 131.5)	5.5 (0.9; 10.4)	481.5 (475.4; 487.4)	384.1 (374.6; 394.6)	− 20.2 (− 22.3; − 17.9)
Piaui	224.8 (208.2; 241.2)	143.7 (137.7; 149.6)	− 36.1 (− 40.6; − 30.6)	24.2 (21.5; 28.5)	13.8 (12.9; 14.9)	− 43.1 (− 51.7; − 34.8)	23.3 (20.8; 26)	27.9 (25.8; 30.1)	20 (5.9; 37.4)	97.2 (88.3; 105)	97.1 (93.3; 101)	− 0.1 (− 8.2; 11)	369.5 (346.5; 390.1)	282.5 (273.1; 291.6)	− 23.5 (− 28.2; − 18.4)
Rio de Janeiro	414 (406.1; 421.4)	196 (188.9; 203.2)	− 52.7 (− 54.5; − 50.9)	45 (42.6; 47.4)	24.4 (22.7; 26.2)	− 45.8 (− 50.3; − 40.8)	44.3 (41.3; 47.4)	30.5 (28.2; 33)	− 31.1 (− 38.1; − 23.5)	178.4 (174.7; 187.1)	147.2 (142.2; 154.9)	− 17.4 (− 20.7; − 14)	681.7 (676.2; 687.3)	398.2 (388.8; 408.6)	− 41.6 (− 43; − 40)
Rio Grande do Norte	166.4 (155.6; 176.5)	136.2 (130.6; 142.2)	− 18.1 (− 24.1; − 11.5)	15.2 (13.7; 16.7)	13.1 (12.1; 14.1)	− 13.7 (− 24.6; − 2.4)	28 (25.4; 30.8)	32.4 (29.8; 35.1)	16 (1.8; 31.8)	100.1 (93.1; 107.3)	119.1 (114.1; 124.1)	18.9 (9.7;31)	309.6 (292.7; 326.5)	300.8 (292.2; 310.2)	− 2.8 (− 8.3;3.2)
Rio Grande do Sul	293.1 (286.8; 299.3)	137.1 (131.6; 142.9)	− 53.2 (− 55.3; − 51)	55.9 (53; 58.9)	30 (28.1; 32.1)	− 46.3 (− 50.7; − 41.6)	20.4 (18.9; 21.9)	19.6 (18; 21.3)	− 3.8 (− 13.6;7.6)	202.7 (198.3; 207)	165.5 (160.4; 171)	− 18.3 (− 21.4; − 15)	572.1 (566.7; 576.7)	352.2 (344; 361.7)	− 38.4 (− 39.9; − 36.8)
Rondônia	308.9 (287.3; 330.9)	146.6 (132.1; 163.8)	− 52.6 (− 58.4; − 46)	45.2 (41.2; 49.6)	25.2 (22.2; 28.5)	− 44.2 (− 52.3; − 34.9)	28.2 (25.5; 31.1)	26 (23; 29.4)	− 7.9 (− 21.1;8.2)	142.6 (131.2; 154.8)	113.9 (102.5; 126.4)	− 20.1 (− 30.1; − 9.7)	524.9 (489.7; 559.9)	311.7 (281.5; 346.2)	− 40.6 (− 47.6; − 33.2)
Roraima	250.1 (228; 273.6)	125.8 (111.5; 142.3)	− 49.7 − 56.8; − 41.5)	29.6 (26.6 ; 33.1)	16.8 (14.6; 19.3)	− 43.5 (− 52.8; − 31.7)	35.2 (31.6; 39)	31.9 (27.7; 36.6)	− 9.2 (− 23.6; 8.9)	141.6 (129.1; 155.3)	115.6 (102.6; 129.5)	− 18.3 (− 29.9; − 4.7)	456.5 (417.5; 497.8)	290.1 (259.8; 325)	− 36.4 (− 45.2; − 26.6)
Santa Catarina	274.9 (268.9; 280.8)	122.2 (117.4; 127)	− 55.6 (− 57.6; − 53.4)	51.8 (49; 54.9)	24.1 (22.4; 25.9)	− 53.4 (− 57.6; − 49)	21.9 (20.3; 23.8)	17.4 (15.9; 18.8)	− 20.8 (− 29.6; − 10.9)	164.6 (161.2; 168.5)	141.8 (137.1; 146.6)	− 13.9 (− 17; − 10.3)	513.3 (508.1; 518.6)	305.5 (297.1; 314.4)	− 40.5 (− 42.2; − 38.7)
São Paulo	321.6 (315; 328.1)	159.4 (153.8; 164.8)	− 50.4 (− 52.4; − 48.5)	36.1 (34.2; 38.2)	21.3 (19.9; 22.8)	− 41 (− 45.8; − 36)	28.3 (26.2; 30.4)	16.2 (14.8; 17.5)	− 42.8 (− 48.9; − 36.2)	168.8 (164.7; 173.3)	138.2 (134.1; 144.3)	− 18.1 (− 21; − 14.8)	554.8 (549.5; 560)	335.1 (326.9; 343.1)	− 39.6 (− 41.1; − 38.1)
Sergipe	196.6 (186.7; 206.3)	144.1 (137.4; 150.9)	− 26.7 (− 31.3; − 22)	25.8 (23.6; 28.3)	19.1 (17.7; 20.6)	− 25.9 (− 33.5; − 17.2)	39.6 (36.2; 43.4)	34.6 (31.9; 37.6)	− 12.5 (− 22.8; − 0.9)	110.4 (105; 115.1)	115.4 (110.9; 120.9)	4.5 (− 1.7; 11.9)	372.4 (362.7; 382.2)	313.2 (301.8; 325.1)	− 15.9 (− 19.5; − 12.5)
Tocantins	236.1 (207; 261.2)	147.4 (137.7; 157.3)	− 37.6 (− 44.2; − 28.5)	29.4 (25.1; 34.1)	16.4 (15; 18)	− 44.2 (− 53; − 33.1)	23.5 (20.2; 26.5)	26.2 (23.9; 28.8)	11.8 (− 4.3; 32.6)	99.6 (86.6; 111.9)	95.8 (89.8; 102.4)	− 3.8 (− 15; 12.1)	388.6 (342.4; 426.3)	285.8 (270.1; 303.1)	− 26.4 (− 33.6; − 16.7)

Legend: Rate of premature mortality by non-communicable diseases (NCD) and main non-communicable diseases (cardiovascular diseases, neoplasms, diabetes, and chronic respiratory diseases) separately standardized by age in the population of individuals between 30 and 69 years of age for both sexes and according to the selected causes of death in Brazil and its states in 1990 and 2017

**Fig. 2:**
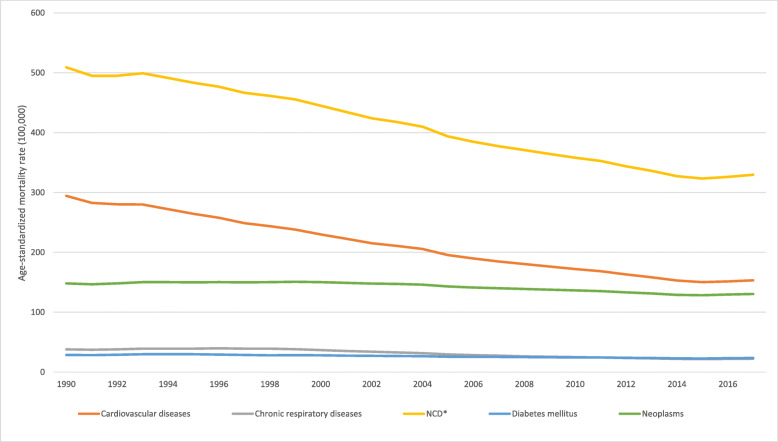
Age-standardized mortality rate by noncommunicable chronic diseases, in the population between 30 and 69 years of age (per 100,000) in Brazil from 2000 to 2017. Legend: Trends of age-standardized mortality rate by non-communicable chronic diseases (yellow line), cardiovascular diseases (orange line), neoplasms (green line), diabetes (blue line), and chronic respiratory diseases (gray line) in the population of individuals between 30 and 69 years of age (per 100,000) in Brazil from 2000 to 2017. *NCD = cardiovascular diseases, neoplasms, diabetes, and chronic respiratory disease

**Fig. 3:**
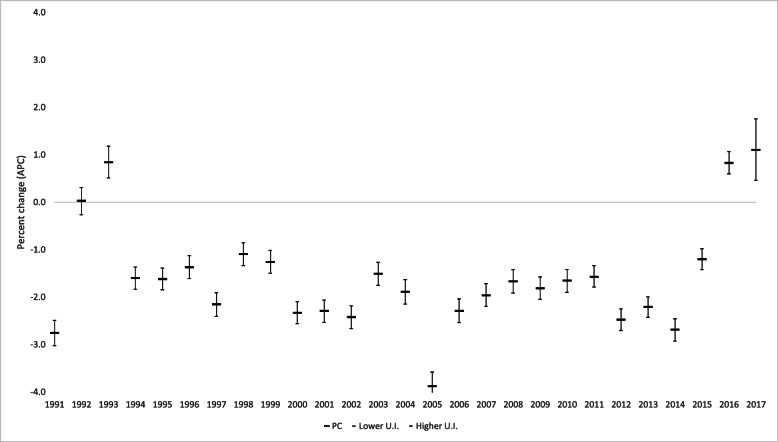
Percentage of annual changes in the age-standardized mortality rate due non-communicable chronic diseases among population between 30 and 69 years of age in Brazil from 1990 to 2017. Legend: Annual percent changes in the age-standardized mortality rate due to noncommunicable chronic diseases (cardiovascular diseases, neoplasms, diabetes, and chronic respiratory diseases) among individuals aged between 30 to 69 years of age in Brazil from 1990 to 2017

Figure 2 shows the age-standardized mortality rates due to all causes and the four groups of non-communicable chronic diseases, which were cardiovascular diseases, cancer, diabetes, and chronic respiratory illnesses, among people aged 30 to 69 years (by 100,000) in Brazil from 2000 to 2017. It was observed that there were 15 continuous years of decline, and in 2015, the trend reversed, with an upward trend in premature deaths from all NCDs and from each NCD group. The annual percent changes in 2016 and 2017 were 1.9% (1.7%; 2.2%) and 2.0% (1.4%; 2.7%), respectively (Fig. 3).

**Fig. 4:**
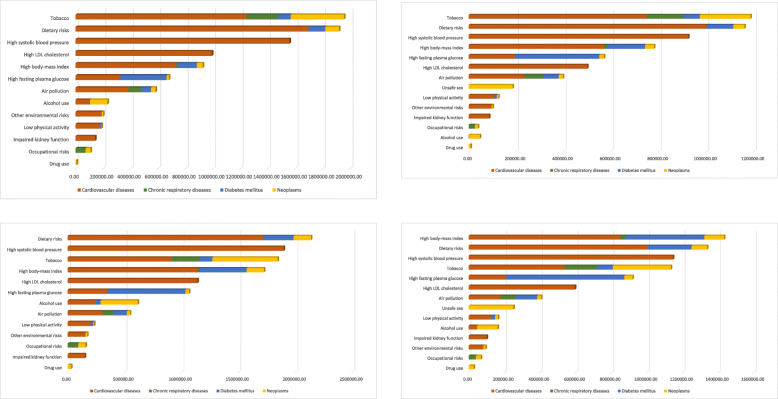
DALYs attributable to risk factors for **a** men and **b** women in the population from 30 to 69 years of age according to the GBD study in Brazil in 1990 and 2017. Legend: DALYs due to cardiovascular diseases (orange bars), chronic respiratory diseases (green bars), diabetes (blue bars), and neoplasms (yellow bars) and attributable to GBD level-2 risk factors for **a** men and **b** women in the population from 30 to 69 years of age according to the GBD study in Brazil in 1990 and 2017

Figure 4 shows the DALYs attributable to the risk factors by NCDs for individuals between 30 and 69 years old in 1990 and 2017 by sex. In 1990, the main RFs that contributed to the DALYs in men were tobacco use, inadequate diet, high blood pressure, and high LDL (low-density lipoprotein) cholesterol. Tobacco use was responsible for approximately 2 million DALYs in 1990, and the main cause of death was cardiovascular diseases, which caused approximately 1.2 million DALYs, and diseases such as neoplasms, chronic respiratory diseases, and diabetes. Among men, the inadequate diet was the most important RF in 2017 and contributed approximately 2.2 million DALYs due to cardiovascular diseases, neoplasms, and diabetes. The other RFs in 2017 were high blood pressure, tobacco use, high body mass index and high LDL cholesterol, high glucose, alcohol use, air pollution, physical inactivity, other environmental and occupational risks, impaired kidney function, and drug use.

In women, the main RFs that contributed to the DALYs in 1990 were tobacco use, inadequate diet, high blood pressure, and high BMI. Tobacco use was responsible for approximately 1.2 million DALYs in 1990. In 2017, high BMI was the highest-ranking RF and contributed approximately 1.5 million DALYs due to CVDs, diabetes, neoplasms, and chronic respiratory diseases. The other RFs in 2017 for women were inadequate diet, high blood pressure, tobacco use, high glucose, LDL cholesterol, air pollution, unprotected sex, physical inactivity, alcohol, impaired kidney function, other environmental and occupational risks, and drug use.

**Fig. 5:**
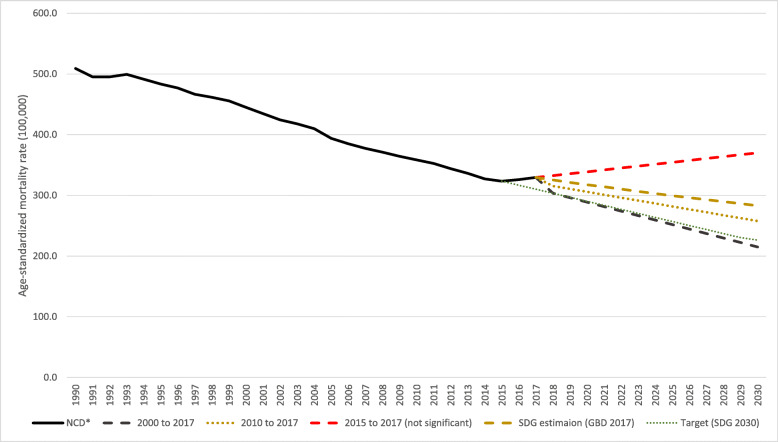
Projections of the rates of premature mortality due to NCD in the Brazilian population for 2030. Legend: Trend (full black line) and projections of age-standardized due to NCD based on 2000 to 2017 (black dashed line), 2010 to 2017 (yellow dotted line), 2015 to 2017 (red dashed line), and SDG (yellow dashes line) estimations and the SDG 2030 target (green dotted line) in the Brazilian population from 30 to 69 years of age in the population for 2030. *NCD = cardiovascular diseases, cancers, diabetes, and chronic respiratory diseases

Figure 5 shows the target proposed by the United Nations in the 2030 Agenda for reducing NCD premature mortality by 30% from 2015 and other projection scenarios for this estimate. Considering the mortality rate in 2015 of 323/100,000 inhabitants, a reduction target of 30% would be 226/100,000 inhabitants in 2030 (dotted lines in Fig. 5). Using the GBD estimates for countries, the projection would be to reach 282/100,000 inhabitants, i.e., achieve a reduction of 13%, below the target of the 2030 Agenda. Using simple linear regression with the rates between 2000 and 2017, the projected rate to 2030 was 215/100,000 inhabitants, a scenario of reaching the target. In the third scenario, with the rates between 2010 and 2017, the projected rate to 2030 was 257/100,000 inhabitants. Finally, using the rates between 2015 and 2017, the result was reverse trend with a projection of increase to 370/100,000 inhabitants in 2030, but it was not significant due to the small number observed points.

## Discussion

The Global Burden of Disease Study showed the accelerated epidemiological transition in the country, with the increase in proportional mortality due to NCDs, which corresponded to around three quarters of total deaths and, of these, a third constituted premature deaths (30 to 69 years of age). A growth in the absolute number of premature deaths from NCDs between 1990 and 2017 and a reduction in the rates of mortality, DALYs and YLLs also occurred in Brazil and most of the states. The more marked reductions were due to cardiovascular diseases followed by chronic respiratory diseases, while diabetes and neoplasms had a pattern that varied by state and a reduction in the whole country. The study showed that the main RFs related to premature deaths from NCDs in 2017 were inadequate diet, high BMI, high blood pressure, and tobacco use. The trends of mortality rates from NCDs declined during the study period; however, after 2015, the curve was inverted, and rates fluctuated and tended to increase, which could compromise the achievement of the SDGs by 2030.

According to the analysis by state, the patterns tended to be similar, such as decreases in premature deaths due to NCDs in most states; however, in relation to diabetes and neoplasms, most states presented increasing or stable trends. We highlighted that the states in the Northeast and North Regions tended to have higher rates, such as Alagoas, Pernambuco, Pará, and Rio Grande do Norte, which has already been demonstrated in previous studies [10, 24]. This might reflect worse life conditions in these regions but could also by due to problems such as low-quality information on mortality, along with a more fragile health system structure and organization in these states, especially in the smaller municipalities, as previously demonstrated [9]. Moreover, the data analyzed here were corrected for underreporting and garbage codes, although the inconsistences in the rates by state might persist.

CVDs and their complications have a high impact on the loss of productivity at work and in the reduction of family incomes [2, 3, 5]. Studies around the world have shown a reduction in the incidence and mortality of CVDs since the 1960s [1]. In Brazil, the decline in CVDs did not occur until the 1990s [25, 26], and the present study confirmed that CVDs declined in the country until 2014, but in recent years, we found an increase. Therefore, it is essential to resume investments in basic healthcare, in addition to investments in regulatory measures and in public policies.

Neoplasms constituted the second leading cause of death in most countries, resulting in approximately 8.9% of global deaths in 2016 [15]. The data from the GBD study show that in 144 countries, there was reduced growth in the age-standardized rates between 2006 and 2016, with most African countries still having increasing rates [15]. In Brazil, the trend was also declining, but the percentage of change was smaller compared to the other NCDs. We should also consider the variations according to the type of cancer, age, and sex. Among women, in the last decades, there was an increase in the mortality rates for breast, lung, and colorectal cancer, and there was a decrease in the mortality rates for cervical and stomach cancer [27]. Among men, there was an increase in the mortality rates for prostate and colorectal cancer and a reduction in those for gastric and lung cancer [27]. The latter was in individuals younger than 60 years of age, which was probably due to the reduction in tobacco usage [27].

Diabetes is associated with increased mortality from CVDs, and their effects are systemic, compromising the cardiovascular system, great arteries, heart, and kidneys [28]. Recent data from the National Health Survey (NHS), which is a household survey carried out in Brazil in 2013 for adults 18 years of age or older, identified that approximately 9.1 million people referred to a previous medical diagnosis of diabetes; in other words, there was a prevalence of 6.2% in all adults [29].

Other authors also showed a reduction in chronic respiratory diseases, which has been attributed to better access to healthcare and medicine and to a decrease in tobacco use [2, 30]. The important decrease in chronic respiratory diseases can be due to the reduction in the prevalence of tobacco use, according to what has already been described in the literature [31].

The Agenda 2030 established the goal of reducing premature mortality from NCDs by 30% through measures of promotion, prevention, and treatment. This goal composes objective 3: “To guarantee healthy lives and to promote wellbeing for everyone in all ages” [4]. Global studies have already shown that health indicators are affected by austerity crises and by an increase in poverty [7]. In Brazil, studies evaluating the impacts of measures of the economic crises and the implementation of Constitutional Amendment 95 in 2016 point to an increase in unemployment, poverty [32], depression [33], and reduced investments in health systems and in the social protection system [33, 34]. As an immediate result, there was an increase in child and maternal mortality [33, 34] and worsening of NCD indicators [8]. More recently, a study has shown the deterioration of four other health indicators in a scenario where SUS funding has been frozen, which highlighted the impact on smaller municipalities, which are highly dependent on federal transfer for the provision of healthcare [9]. Thus, this long-term fiscal austerity policy could also increase geographical inequality, such as at the state level, including preventable NCD mortality, which might contribute to reversing the recent trend of overall improvement [9].

This elevated burden of NCDs arose from some negative effects of the globalization process, social inequalities, poverty, and accelerated urbanization, which resulted in an increase in physical inactivity and high caloric value nutrition in addition to consumption of tobacco and alcohol [1, 2]. These behavioral risk factors impacted the main metabolic risk factors and consequently the increase of NCD [1, 14, 35]. Evidence shows that the control of RFs and NCDs is more effective when regulatory measures imposed by the states interfere with the environment and regulate commercial practices, availability, and service offers [35]. Some of the best evidence shows the benefits of the taxation of products that are injurious to health, food labeling, regulation of tobacco and alcohol products [35], government measures that establish limits for the use of salt by industries [36], among other actions [37].

This study also pointed out the decrease in tobacco use as an RF for NCDs in both sexes between 1990 and 2017. This could be explained by the many regulatory measures that were adopted, while the country was considered an example for the world in the reduction of the prevalence of tobacco use [38]. Among these measures, we highlighted the prohibition of tobacco propaganda through the ratification of the Framework-Convention on Tobacco Control (FCTC) in 2006 and Federal Law n^o^ 12.546 in 2011. We also highlight the presidential decree from 2014 that instituted spaces free of tobacco, an increase in the size of cigarettes warnings, an increase in cigarettes taxation, and the definition of a minimum price for its commercialization, among others [39].

However, in recent years, an increase in the consumption of other tobacco products has been observed, especially hookah among adolescents [40], less inspection of tobacco products, no readjustment of cigarette prices, and an increase in illegal commerce [8, 41], which might impact consumption. New regulatory measures must be implemented, such as adopting generic packaging for tobacco products, applying inspection for tobacco-free spaces, and the points of sale, preventing the illegal commerce from smuggling and investing in the support of small farmers in the diversification of the cultures, among other strategies [35, 42].

An inadequate diet was the most prominent RF for men in 2017, and high BMI was the first for women, which pointed out the urgency of investing in policies to promote healthy foods, such as fruits and vegetables, and to regulate ultra-processed foods [35] in addition to incentivizing physical activity [35, 37].

As identified above, obesity constitutes a large problem in the country and has tended to increase, reaching 24% of women in 2013 according to the PNS data [42, 43]. The challenges to stopping the increase in this problem are great and require effective regulatory measures; the approval of legislation about the taxation of ultra-processed foods; subsidies for healthy foods; and the prohibition of the marketing food to children [34]. Measures such as the taxation of sweetened beverages are among the recommendations currently made by the WHO and have already been adopted in many countries, such as Mexico, which experienced a 10% reduction in soft drink consumption [35, 37]. However, facing this problem will require the political will and the confrontation of food industry interests [44].

In the last decade, important measures have been adopted by the Ministry of Health, such as in 2011 with the launch of the Strategic Action Plan for Tackling Chronic Non-Communicable Diseases [11], which advocated shopping for fresh foods from small farmers and the distribution of these goods in schools [45]; the Food Guide of the Brazilian Population (2014) [46] in 2014; the promotion of incentives for breastfeeding, and the voluntary agreement for the reduction of salt in ultra-processed foods [44]. This agenda, however, needs to be enhanced so that we advance further in tackling NCDs in Brazil. Confronting NCDs implies managing metabolic risks and, therefore, adequate functioning of primary healthcare, in addition to access to medium- to high-complexity technology, when necessary, to increase the integral care to the NCD patients, including the management of high blood pressure and diabetes [1, 25, 47]. The NCDs require a longitudinal approach in their management, with investments in self-care and in the accountability of the healthcare teams so that effective results can be achieved [5]. The SUS serves most of the Brazilian population, and more than 60% of the population relies on the primary healthcare coverage [9]. In the last decade, important programs for primary care access have been implemented, such as the Family Health Strategy; the Brazilian Program *Mais Médicos* (2013), which provided free access to NCD medications; *Aqui tem Farmacia Popular*, which assisted in paying for medicines since 2004, and since 2011, with a free group of medicines for NCDs [48]; the NCDs Plan; and access to cash transfer programmes (2003) [13, 49], which explained the decrease in mortality rates by NCDs until 2014.

However, after 2015, the political and economic crisis resulted in the reduction in investments in public policies and the increase of unemployment and poverty, which could explain the worsening of the NCDs indicators [8, 32, 33]. As many studies showed, NCDs are concentrated in more vulnerable populations with low income and low education levels [3, 50, 51], which are sensitive to factors such as economic crisis and austerity measures [6–8, 33]. Therefore, to achieve the SDGs, it is necessary to search for equity, for access of vulnerable populations to be widened, and for investments to me made in SUS (Brazil’s unified health system) and in the social inclusion policies [33, 34]. The strategy of austerity measures and budget cuts will prevent the achievement of SDGs in the country [33, 34].

SUS has been able to protect universal access for most of the population [49–52]. However, Constitutional Amendment 95, which was approved in December 2016, established that the budgets for health, education, science, technology, and other social policies shall remain stable for the next 20 years, which could represent a serious threat to the access and to the completeness of healthcare and to NCD patients who require continuity and quality of care [52].

In addition, we can highlight that the implementation of NCD surveillance in the country after 2003 was fundamental for identifying these trends and the NCD risk factors, such as the important modifications in trends in recent years, the increase in alcohol consumption, the worsening of healthy eating indicators [8, 53]. The maintenance of continuous surveillance has become crucial for monitoring the SDGs and identifying the impacts of certain policies regarding the population’s health, such as the negative effects of the austerity policies [33, 34].

There were limitations in the data source that was used. Although SIM has widened the cover of registries and improved its quality in recent years, in the past and still in some states, there are unregistered deaths, incomplete registries, and a high proportion of *garbage codes.* Studies have alerted to the need for SIM correction and have advocated adjustment methodologies, such as the active search for deaths and the investigation of deaths [5]. Due to these problems, the GBD study performs statistical procedures to correct the estimates produced, which allowed geographic and time comparisons. In addition, the GBD study assumed that the relative risks were even in all countries for a certain age and sex, which cannot be correct for all countries, and this could affect the results [13].

## Conclusion

Our study revealed that despite the progressive decrease during the first years, a change in the trends of premature mortality rates due to NCDs occurred in the last 3 years; therefore, the goal of reducing NCD mortality by 30% by 2030 could not be achieved.

The study also shows the important contribution and the profile change of the behavioral RFs implicated in the mortality from NCDs, such as inadequate diet, tobacco use, physical inactivity, alcohol use, and the metabolic factors resulting from them (high BMI, high blood pressure, glucose, and high cholesterol).

We considered that the adopted austerity policies, the economic crisis, and the increase in poverty tended to be aggravated with the implementation of measures such as budget cuts for health due to Constitutional Amendment 95. Therefore, the country will need to boost its efforts to achieve the goal, which points to a complex and pessimistic scenario. Thus, such findings draw attention to the consequences of austerity measures in socially unequal settings with great regional disparities in which the majority of the population is dependent on state social policies.

These data support the country in the monitoring of the SDGs, which constitute the most visible and highest priority global agenda.

## Data Availability

All data that we used in this article are publicly available online on the official website of the Institute of Health Metrics and Evaluation (http://ghdx.healthdata.org/gbd-results-tool).

## Abbreviations

IBGE: Brazilian Institute of Geography and Statistics
CVD: Cardiovascular diseases
DALYs: Disability-adjusted life years
COEP/UFMG: Ethics Committee in Research of the Federal University of Minas Gerais
FD: Federal District
FCTC: Framework-Convention on Tobacco Control
GBD: Global Burden of Disease
BMI: High body mass index
SIM: Information System about Mortality
IHME: Institute of Health Metrics and Evaluation
LDL: Low-density lipoprotein
LMICs: Low- and middle-income countries
NHS: National Health Survey
PeNSE: National Student Health Survey
NCDs: Non-communicable diseases
RF: Risk factors
SUS: Sistema Único de Saúde
SDGs: Sustainable Development Goals
VIGITEL: Telephone survey surveillance system for risk and protective factors for chronic diseases
UI: Uncertainty intervals
WHO: World Health Organization
YLLs: Years of life lost
